# The effect of potent CYP2D6 inhibition on the pharmacokinetics and safety of deutetrabenazine in healthy volunteers

**DOI:** 10.1007/s00228-021-03202-0

**Published:** 2021-09-01

**Authors:** F. Schneider, D. Stamler, M. J. Bradbury, P. S. Loupe, M. F. Gordon, L. Rabinovich-Guilatt

**Affiliations:** 1 Teva Pharmaceutical Industries Ltd, Ratiopharm GmbH, Ulm, Germany; 2 Formerly of Teva Pharmaceutical Industries Ltd, Currently Alterity Therapeutics Limited, Newark, CA USA; 3 Teva Pharmaceutical Industries Ltd, West Chester, PA USA; 4 Teva Pharmaceutical Industries Ltd, Netanya, Israel

**Keywords:** Deutetrabenazine, Tetrabenazine, Deuteration, CYP2D6 inhibition

## Abstract

**Purpose:**

Deutetrabenazine is a deuterated form of tetrabenazine with a confirmed lower rate of CYP2D6 metabolism of the active metabolites, α- and β-HTBZ. In this study, we assessed the effect of paroxetine, a potent CYP2D6 inhibitor, on the pharmacokinetics and safety of deutetrabenazine and its metabolites.

**Methods:**

In this open-label sequential drug-drug-interaction study, 24 healthy adults who were CYP2D6 extensive or intermediate metabolizers received a single deutetrabenazine 22.5-mg oral dose on days 1 and 11 and a single paroxetine 20-mg oral daily dose on days 4–12. Pharmacokinetics of deutetrabenazine and its metabolites were assessed on days 1–4 and 11–14. Paroxetine trough concentrations were obtained pre-dose on days 9–13. Safety examinations occurred throughout the study.

**Results:**

Paroxetine administered under steady-state conditions, increased exposure of the deuterated active metabolites, α-HTBZ (1.2-fold C_max_ and 1.8-fold AUC_0–∞_) and β-HTBZ (2.1-fold C_max_ and 5.6-fold AUC_0–∞_), and correspondingly, 1.6-fold C_max_ and threefold AUC_0–∞_ for total (α + β)-HTBZ. Sixteen subjects reported 45 adverse events and most were mild. Headache was the most common AE reported 8 times by 7 subjects (5 following paroxetine alone; 2 following deutetrabenazine + paroxetine).

**Conclusions:**

Paroxetine-induced increases in exposure to the active deutetrabenazine metabolites were less than those previously reported for tetrabenazine, a finding expected to reduce the burden of drug interaction. In addition, single doses of 22.5 mg deutetrabenazine, when given alone or in the presence of steady-state paroxetine (20 mg daily), were safe.

**Supplementary information:**

The online version contains supplementary material available at 10.1007/s00228-021-03202-0.

## Introduction

Deutetrabenazine (Austedo, Teva Pharmaceutical Industries Ltd) and tetrabenazine (Xenazine, Lundbeck) are approved to treat chorea associated with Huntington’s disease [1, 2]. Deutetrabenazine is also approved to treat tardive dyskinesias [3, 4]. Both compounds, through their active α- and β-dihydrotetrabenazine (HTBZ) metabolites, function as vesicular monoamine transporter 2 (VMAT2) inhibitors, a class of drugs that acts by depleting presynaptic monoamines, in particular dopamine, in the basal ganglia.

Deutetrabenazine was rationally designed as a deuterated form of tetrabenazine in which two O-linked methyl groups (O–CH3) of the tetrabenazine molecule have been replaced by two trideuteromethyl groups (O-CD3). Studies in healthy volunteers confirmed that the deuteration at the selected positions attenuates the metabolic breakdown of α-HTBZ and β-HTBZ by CYP2D6 with corresponding reductions in the formation of the less active O-demethylated metabolites, thus leading to doubling of the active metabolite half-lives without formation of novel metabolites [5, 6]. A schematic showing deutetrabenazine metabolism from Schneider et al. 2020 is shown as Supplemental Fig. S1 [5]. As a consequence, deutetrabenazine can be administered at lower doses relative to tetrabenazine to achieve a comparable systemic exposure to the active α- and β-HTBZ metabolites with a reduction in the maximal concentration (C_max_). The increased half-life and lower C_max_ result in reduced peak-to-trough fluctuations of the active metabolites at steady state compared to tetrabenazine. Further, the longer half-life has led to twice daily dosing of deutetrabenazine in all dosing regimens [1, 3, 4] whereas tetrabenazine may require up to three times daily dosing [7, 8]. Strong CYP2D6 inhibitors, such as paroxetine, are known to significantly increase plasma levels of α- and β-HTBZ after tetrabenazine administration [7, 9]. Based on this interaction, recommended doses of tetrabenazine in patients taking strong CYP2D6 inhibitors or those with poor CYP2D6 metabolism phenotypes must be reduced. Since deuteration attenuates the CYP2D6 metabolism of α- and β-HTBZ, it was predicted that the effect of a strong CYP2D6 inhibitor on these metabolites would be lower for deutetrabenazine than for tetrabenazine. Thus, in this study, the pharmacokinetic profiles of deutetrabenazine and its metabolites were assessed in the absence and presence of paroxetine, a potent CYP2D6 inhibitor. Paroxetine is commonly used in drug-drug interaction studies as a representative potent CYP2D6 inhibitor and is also a frequent concomitant medication in the intended patient population. The quantification of the impact with deuteration in exposure in patients with impaired CYP2D6 function was used, together with population pharmacokinetic modeling (data on file, Teva Pharmaceutical Industries, Ltd), to support appropriate dosing recommendations for deutetrabenazine for registration studies.

## Methods

### Ethics

The study was performed in accordance with Good Laboratory Practice and the International Conference on Harmonization Harmonized Tripartite Guideline regarding Good Clinical Practice. The study was conducted by Celerion in Tempe, Arizona, USA (Principal Investigator, Terry O’Reilly, MD, Certified Physician Investigator), and all study documents were reviewed by the Institutional Review Board, Chesapeake Research Review, Inc. The study protocol was designed by Auspex Pharmaceuticals (La Jolla, CA, USA), which was acquired by Teva Pharmaceutical Industries Ltd (Petah Tikva, Israel).

### Study participants

This study included healthy male or female volunteers, between 18 and 50 years of age. Subjects were excluded if they were a CYP2D6 poor (null/null genotype) or ultra-rapid (3 or more wild type alleles) metabolizer. Other exclusion criteria were the use of tobacco or nicotine products in the previous 3 months, use of any over-the-counter medications, herbal or hormone supplements other than approved hormonal contraception, or diet aids within 14 days of start of dosing. Approved doses of acetaminophen or ibuprofen were not excluded.

### Study design

This was an open-label sequential drug-drug interaction study in 24 healthy adult subjects who were CYP2D6 extensive metabolizers (EM) or CYP2D6 intermediate metabolizers (IM); Supplemental Table S1 provides the genotypes and phenotypes of the subjects. The genotype testing was performed by Genelex (Seattle, WA, USA) on a Luminex 100xMAP IS system using the xTAGCYP2D6 v2 assay kit (Luminex Molecular Diagnostics Inc, Toronto CA) which detects a panel of 17 small nucleotide variants found within the highly polymorphic cytochrome P450-2D6 gene located on chromosome 22, gene rearrangements associated with the deletion (*5) and duplication genotypes.

Subjects checked into the clinic on day 1 and were confined through day 14. While confined in the clinic, subjects were given frequent safety assessments, including adverse event reporting, ECG recordings, and physical and laboratory examinations including the Columbia Suicide Severity rating scale (C-SSRS), an FDA-mandated questionnaire to screen for suicidality in trials of CNS active compounds [10]. Those who met inclusion/exclusion criteria received a single dose of deutetrabenazine on day 1 followed by a 72-h washout. Subjects then received paroxetine on days 4 to 12 to achieve steady-state plasma concentrations of paroxetine by day 11. On day 11, another single dose of deutetrabenazine was administered. Triplicate 12-lead digital electrocardiograms (ECGs) were collected predose and for 10 h following deutetrabenazine dosing on day 1, and for 10 h on day 11, following paroxetine administration on day 10. Blood samples for measurement of plasma concentrations of deutetrabenazine and its metabolites were obtained on days 1 and 11 (predose, 1, 2, 2.5, 3, 3.5, 4, 4.5, 5, 6, 7, 8, 10, 12, and 16 h), days 2 and 12, at 24 and 36 h, days 3 and 13, at 48 and 60 h and days 4 and 14, at 72 h. Paroxetine trough levels were obtained pre-dose on days 9, 10, 11, and 12, and on day 13 at 24 h post the final paroxetine dose on day 12. Blood and urine samples were collected for clinical laboratory testing (hematology, serum chemistries, and urinalysis) at admission and after at least 10 h fasting and at study completion on day 14 or upon early withdrawal.

## Treatments

All participants received the following treatments, deutetrabenazine 22.5-mg oral dose as one 15-mg tablet + one 7.5-mg tablet on days 1 and 11 and one paroxetine 20 mg (Cadila Healthcare Ltd.) oral tablet on days 4 through 12. The deutetrabenazine 22.5-mg dose was administered with 237 mL of water in the morning approximately 30 min after a standardized breakfast meal with moderate fat content (35%). No food was allowed for at least 4 h postdose, and subjects were asked to remain upright for at least 4 h postdose, unless medically necessary.

Paroxetine 20 mg was administered in the morning with 237 mL water for 9 days (days 4 to 12) to ensure steady-state levels. On days 4 to 10 and 12, the paroxetine dose was administered in a fed state 30 min after breakfast. On day 11, as paroxetine can be administered without regard to food [11], subjects took the paroxetine dose under fasting conditions (at least 1 h prior to breakfast and 1.5 h prior to deutetrabenazine administration) following an overnight fast of at least 10 h. Subjects were asked to remain upright for at least 1 h after the paroxetine dose, unless medically necessary.

### Bioanalytical measures

Validated liquid chromatography/tandem mass spectrometry methods were applied to assess plasma concentrations of paroxetine, deutetrabenazine, α- and β-HTBZ metabolites, and the O-desmethyl metabolites of α- and β-HTBZ. This was performed on a Sciex API 4000 instrument, equipped with a Turbo Ion Spray ion source operated in the positive ion mode and interfaced to a Shimadzu HPLC system fitted with a Waters XBridge C_18_ reverse phase column (3.5 µm; 50 × 2.1 mm) for paroxetine, deutetrabenazine, α-HTBZ and β-HTBZ or a Phenomenex Kinetex column (2.6 µm; 100 × 2.1 mm) for the O-desmethyl metabolites. The validated assay ranges were as follows: paroxetine 0.500 to 50.0 ng/mL, deutetrabenazine 0.100 to 10.0 ng/mL, deuterated α- and β-HTBZ 0.500 to 100 ng/mL, and 9-O- and 10-O-desmethyl metabolites of α-HTBZ and β-HTBZ 0.5 to 50.0 ng/mL. The methods were developed and validated by CPR Pharma Services, Pty, Ltd Australia, as described earlier [5].

### Pharmacokinetic analyses

Plasma concentrations, below the limit of quantitation (BLQ) of the assay, were designated a value of zero for initial and time points occurring at the end of the profile. For sampling time points between two quantifiable concentrations, plasma concentrations BLQ followed by a measurable concentration were set to the limit of quantitation of the assay. Total deuterated (α + β)-HTBZ was calculated as the sum of the α- and β-HTBZ metabolites. The imputed value was used for calculation of total deuterated (α + β)-HTBZ, for calculation of descriptive summary statistics, in determination of PK parameters, and for presentation in the individual profiles. The PK parameters were determined using Phoenix WinNonlin, Version 6.3 (Pharsight Corporation). PK parameters included the following: area under the plasma concentration–time curve from time 0 to the time of the last quantifiable concentration (AUC_0−*t*_), area under the plasma concentration–time curve from time 0 to infinity (AUC_0–∞_), maximum observed plasma drug concentration (C_max_), time to C_max_ (T_max_), terminal elimination half-life (*t*_1/2_), terminal elimination rate constant (*λ*_*Z*_), percentage of AUC_0–∞_ extrapolated (%AUC_extrap_), apparent total body clearance (CL/*F*), and apparent volume of distribution (*V*_*z*_/*F*).

### Statistical analysis

No formal sample size calculation was performed. The sample size was selected based on the magnitude of effect observed in a previous study looking at the effect of paroxetine on the pharmacokinetic of tetrabenazine, where a threefold increase in the exposure of α-HTBZ and a ninefold increase in the exposure of β-HTBZ were observed [7]. It was anticipated that 24 subjects would be sufficient to characterize this effect and allow recommendations on doses of deutetrabenazine to be used in combination with CYP2D6 inhibitors.

The effect of paroxetine on the pharmacokinetics of deutetrabenazine was assessed using two-sided 90% confidence intervals (CIs) for the differences between log-transformed values of the parameters C_max_, AUC_0−*t*_, and AUC_0–∞_. Confidence intervals (90%) were constructed for the treatment ratios (deutetrabenazine + paroxetine to deutetrabenazine alone) of C_max_, AUC_0−*t*_, and AUC_0–∞_ for α-HTBZ, β-HTBZ, and total (α + β)-HTBZ and O-desmethyl HTBZ metabolites as required using the log-transformed data. The point estimates and confidence limits were back-transformed to the original scale. The attainment of steady state with respect to paroxetine was assessed by comparing the predose plasma concentrations on treatment days 9, 10, 11, 12, and 13 (24 h after the final paroxetine dose on day 12) graphically. Descriptive statistics were calculated for plasma concentration–time profiles and pharmacokinetic parameters.

## Results

### Subject disposition and demographics

All 24 enrolled subjects (16 EM and 8 IM participants) completed the study. One subject could not be correctly classified regarding his CYP2D6 metabolizer status because of multiple copies of one of the alleles which could be classified as an ultra-metabolizer, meeting an exclusion criterion and thus was excluded from the primary PK analysis population; however, his data were included in a secondary analysis of bioavailability. The mean (SD) age of participants was 31.6 (9.8) years, and mean (SD) weight was 70.5 (11.3) kg; 33% were female, and 96% were white, and 1 (4%) was black or African American.

### Safety

Single doses of 22.5 mg deutetrabenazine, when given alone or in the presence of steady-state paroxetine (20 mg daily), were safe and generally well tolerated in healthy subjects. There were no deaths, serious adverse events, cardiac safety events, suicidal behavior (based on C-SSRS), or discontinuations due to adverse events. A summary of the proportion of subjects experiencing AEs is presented in Supplemental Table S2. Most TEAEs were mild in severity and resolved without sequelae. All mean ECG parameters (HR, PR, QRS, RR, QTc, and T-wave amplitude) remained within normal limits, with QTcF mean intervals remaining < 410 ms following all treatments.

#### Pharmacokinetic evaluations

**Fig. 1:**
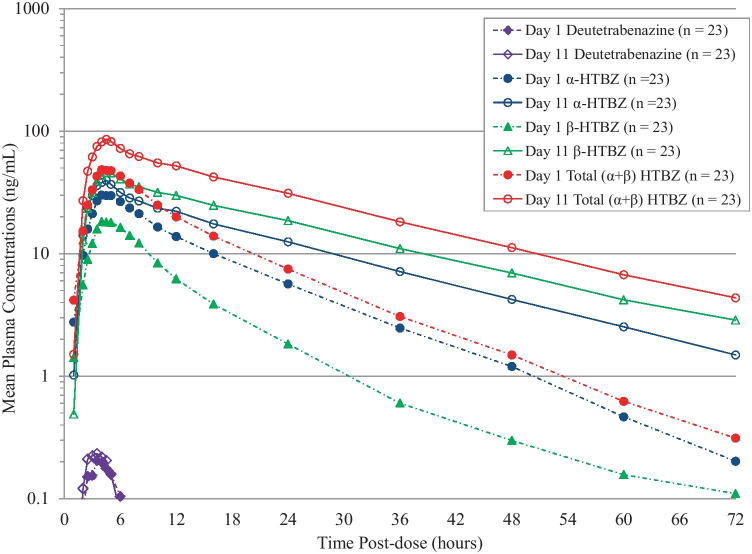
Mean plasma concentrations of deutetrabenazine and primary metabolites in the absence (dose day 1) and presence (dose day 11) of paroxetine. Deutetrabenazine 22.5 mg was orally administered on days 1 and 11. Paroxetine 20 mg was orally administered on days 4–12. Plasma sampling for assessment of deutetrabenazine and α-HTBZ and β-HTBZ concentrations occurred for 72 h after each dose. HTBZ dihydrotetrabenazine

Steady-state exposure of the strong CYP2D6 inhibitor paroxetine was achieved on day 10 as confirmed by visual inspection of the mean trough plasma concentrations. The mean plasma concentrations of deutetrabenazine and the active metabolites (α-HTBZ, β-HTBZ, and total (α + β)-HTBZ) in the absence (day 1) and presence (day 11) of steady-state concentrations of paroxetine are presented in Fig. 1. The parent drug deutetrabenazine appeared only transiently in plasma, and its concentrations were remarkably lower than concentrations of the deuterated active metabolites. Mean plasma concentrations of deutetrabenazine in the presence and absence of paroxetine were comparable. Mean plasma concentrations of the active metabolites α-HTBZ and β-HTBZ individually and in total were higher in the presence of paroxetine compared to the same subjects receiving deutetrabenazine alone.

**Fig. 2:**
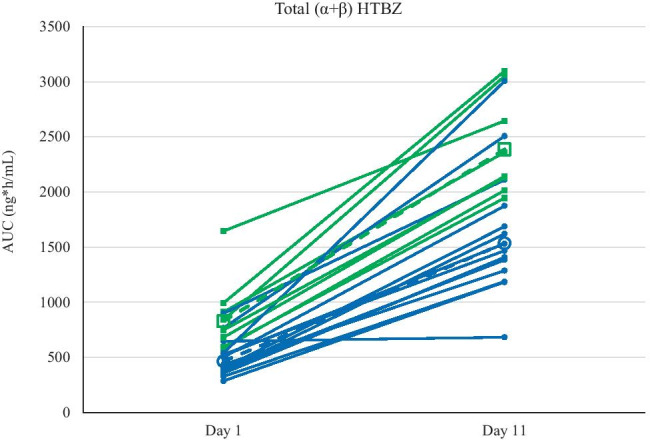
Individual AUC_0−∞_ (ng × h/mL) values for each subject for total (α + β) dihydrotetrabenazine following deutetrabenazine dosing in the absence (dose day 1) and presence (dose day 11) of paroxetine. Deutetrabenazine 22.5 mg was orally administered on days 1 and 11. Paroxetine 20 mg was orally administered on days 4–12. AUC_0−∞_ values for subjects with CYP2D6 extensive metabolizer phenotype are shown as filled blue circles and blue lines; geometric mean is shown as an open circle with a dashed blue line. AUC values for subjects with CYP2D6 intermediate metabolizer phenotype are shown as filled green squares with green lines; geometric mean is shown as an open square with a dashed green line

Key PK parameters of the parent drug and the deuterated active metabolites in the absence and presence of paroxetine, overall and by phenotype (EM or IM), are provided in Table 1. The strong CYP2D6 inhibitor paroxetine increased C_max_ and total exposure (AUC_0–∞_) of both metabolites. The increased exposure was associated with an increased half-life (*t*_1/2_) of both active metabolites with a stronger effect on β-HTBZ (2.7-fold) versus α-HTBZ (1.5-fold). The extent of increase in presence of paroxetine was higher for β-HTBZ (2.1-fold for C_max_ and 5.6-fold for AUC_0–∞_) compared to α-HTBZ (1.2-fold for C_max_ and 1.8-fold for AUC_0–∞_) and higher for total exposure versus maximum exposure. The higher exposure of the individual metabolites led to an about 1.6-fold higher C_max_ and threefold higher AUC_0–∞_ for total (α + β)-HTBZ in a population of 15 EM and 8 IM (Fig. 2). The effect of paroxetine was observed to be greater in EM phenotype subjects compared with subjects with IM phenotype. In comparison to EM, the observed changes in IM were about 30 and 20% lower for C_max_ and AUC_0–∞_, respectively. For comparisons of C_max_, AUC_0–*t*_, and AUC_0-∞_ in the presence and absence of paroxetine, % ratios of LS means and corresponding 90% confidence intervals are provided in Table 2.

**Table 1:** Summary of key pharmacokinetic parameters of primary analytes by dose day and CYP2D6 phenotype

Day 1 = deutetrabenazine alone Day 11 = deutetrabenazine + Paroxetine Mean (CV%)	All Subjects (*n* = 23)		Subjects with phenotype EM (*n* = 15)		Subjects with phenotype IM (*n* = 8)	
	Dose day 1	Dose day 11	Dose day 1	Dose day 11	Dose day 1	Dose day 11
Deutetrabenazine						
C_max_ (ng/mL)	0.4 (60)	0.4 (53)	0.3 (44)	0.4 (57)	0.5 (66)	0.4 (49)
T_max_ (h)^a^	3.5 (1.0–6.0)	3.0 (2.0–4.5)	3.5 (1.0–6.0)	3.0 (2.0–4.5)	3.5 (1.0–6.0)	3.3 (2.0–4.5)
AUC_0-t_ (ng × h/mL)	0.8 (53)	0.8 (60)	0.6 (40)	0.7 (67)	1.0 (55)	1.0 (50)
*t*_1/2_ (h)^b^	NC	NC	NC	NC	NC	NC
α-HTBZ						
C_max_ (ng/mL)	37.4 (22)	45.4 (25)	35.8 (21)	46.6 (27)	40.5 (22)	43.2 (21)
T_max_ (h)^a^	4.0 (2.0–7.0)	3.5 (2.0–5.0)	4.0 (2.0–6.0)	3.5 (2.0–4.5)	4.0 (2.0–7.0)	3.5 (2.5–5.0)
AUC_0–∞_ (ng × h/mL)	427 (34)	780 (28)	363 (31)	706 (29)	546 (22)	921 (18)
*t*_1/2_ (h)	10.5 (28)	15.5 (18)	9.1 (20)	14.0 (14)	13.0 (23)	18.1 (11)
β-HTBZ						
C_max_ (ng/mL)	24.6 (38)	51.5 (25)	20.4 (33)	52.0 (28)	32.6 (22)	50.5 (21)
T_max_ (h)^a^	4.01 (2.0–8.0)	3.6 (2.0–6.0)	4.0 (2.0–6.0)	3.5 (2.0–5.0)	4.0 (2.0–8.0)	4.3 (3.5–6.0)
AUC_0–∞_ (ng*h/mL)	199 (90)	1121 (40)	128 (46)	916 (40)	333 (75)	1507 (20)
*t*_1/2_ (h)	5.9 (48)	16.2 (28)	4.8 (24)	13.7 (19)	7.9 (50)	21.1 (15)
Total (α + β) HTBZ						
C_max_ (ng/mL)	61.8 (26)	95.9 (25)	56.0 (25)	97.8 (27)	72.8 (19)	92.2 (21)
T_max_ (h)^a^	4.0 (2.0–8.0)	3.6 (2.0–6.0)	4.0 (2.0–8.0)	3.5 (2.0–5.0)	4.3 (2.0–8.0)	4.3 (2.5–6.0)
AUC_0–∞_ (ng*h/mL)	624 (49)	1901 (34)	489 (35)	1622 (35)	874 (39)	2423 (19)
*t*_1/2_ (h)	9.8 (28)	16.0 (24)	8.6 (23)	14.1 (20)	12.0 (23)	19.8 (13)

*NC* not calculated, *AUC*_*0–∞*_ the area under the plasma concentration versus time curve from time 0 extrapolated to infinity, *C*_*max*_ maximum plasma concentration; *CV* coefficient of variation, *EM* extensive metabolizer, *HTBZ* dihydrotetrabenazine, *IM* intermediate metabolizer, *t*_*1/2*_ half-life, *T*_*max*_ maximum plasma concentration

^a^Median (min–max)

^b^Half-life could not be calculated for the parent drug, deutetrabenazine because the terminal elimination rate constant could not be determined

**Table 2:** Comparison of pharmacokinetic parameters by analytes for deutetrabenazine administration with and without paroxetine administration

Day 1 = deutetrabenazine alone Day 11 = deutetrabenazine + paroxetine		% Ratio of LS means (90% CI) (day 11)/(day 1)		
		Subjects with EM phenotype (*n* = 15)	Subjects with IM phenotype (*n* = 8)	All subjects (*n* = 23)
α-HTBZ	C_max_ (ng/mL)	119.9 (108.1–132.9)	127.8 (111.8–146.1)	106.3 (90.0–125.4)
	AUC_0−t_ (ng × h/mL)	181.6 (162.4–302.0)	192.2 (162.9–226.8)	163.3 (148.3–179.8)
	AUC_0−∞_ (ng × h/mL)	185.0 (165.8–206.4)	193.6 (164.4–228.0)	169.8 (153.8–187.5)
β-HTBZ	C_max_ (ng/mL)	216.4 (185.0–253.2)	259.2 (216.7–310.1)	154.3 (126.0–189.0)
	AUC_0−t_ (ng × h/mL)	641.3 (537.3–765.4)	744.6 (605.3–915.8)	484.7 (355.2–661.5)
	AUC_0−∞_ (ng × h/mL)	649.9 (549.6–768.5)	731.5 (595.0–899.3)	520.6 (388.9–697.0)
Total (α + β)-HTBZ	C_max_ (ng/mL)	154.8 (137.4–174.4)	173.1 (149.8–200.1)	125.6 (105.4–149.6)
	AUC_0−t_ (ng × h/mL)	303.6 (268.0–343.8)	324.9 (273.9–385.4)	267.3 (224.0–318.9)
	AUC_0−∞_ (ng × h/mL)	314.7 (278.4–355.6)	329.8 (277.7–391.8)	288.1 (242.3–342.5)

*C*_*max*_ maximum plasma concentration, *AUC*_*0−t*_ area under concentration–time curve from time 0 to time of the last quantifiable concentration, *AUC*_*0−∞*_ the area under the plasma concentration versus time curve from time 0 extrapolated to infinity, *HTBZ* dihydrotetrabenazine, *LS* means (90% CI) least-square means with 90% confidence intervals, *EM* extensive metabolizer, *IM* intermediate metabolizer

Prolongation of the half-lives of α-HTBZ and β-HTBZ in the presence of paroxetine was associated with reduced production of O-desmethyl metabolites of HTBZ. Briefly, 9-O-desmethyl α- and β-HTBZ were quantifiable throughout the time course while 10-O-desmethyl α-HTBZ was below the lower limit of quantitation on all-time points and 10-O-desmethyl β-HTBZ appeared transiently in some subjects in the absence of paroxetine and was below the lower limit of quantitation in the presence of paroxetine indicating a reduced formation of this metabolite. PK parameters of 9-O-desmethyl α-HTBZ and 9-O-desmethyl β-HTBZ are displayed in Table 3. In the overall population, AUC_0−t_ and C_max_ were lower by a factor of 0.3 and 0.4 for 9-O-desmethyl α-HTBZ and 0.7 and 0.4 for 9-O-desmethyl β-HTBZ, respectively.

**Table 3:** Summary of key pharmacokinetic parameters of 9-O-desmethyl HTBZ metabolites overall and by CYP2D6 phenotype

Dose day 1 = Deutetrabenazine alone Dose day 11 = Deutetrabenazine + paroxetine Mean (CV%)	All participants (*n* = 23)^a^		Subjects with phenotype EM (*n* = 15)^b^		Subjects with phenotype IM (*n* = 8)^c^	
	Dose day 1	Dose day 11	Dose day 1	Dose day 11	Dose day 1	Dose day 11
9-O-desmethyl α-HTBZ						
C_max_ (ng/mL)	1.3 (28)	0.5 (73)	1.4 (18)	0.5 (57)	1.2 (45)	0.4 (112)
T_max_ (h)^d^	5.0 (3.0–12.0)	4.5 (3.5–24.1) (*n* = 16)	5.0 (3.0–8.0)	4.5 (3.5–10.0) (*n* = 12)	5.5 (3.5–12.0)	4.3 (4.0–24.1) (*n* = 4)
AUC_0–t_ (ng × h/mL)	13.9 (48)	4.6 (128)	13.9 (39)	4.3 (122)	13.8 (65)	5.1 (144)
*t*_1/2_ (h)	NC	NC	NC	NC	NC	NC
9-O-desmethyl β-HTBZ						
C_max_ (ng/mL)	4.4 (29)	1.6 (36)	4.5 (20)	1.6 (31)	4.2 (45)	1.6 (47)
T_max_ (h)^a^	6.0 (2.5–24.0)	16.0 (4.5–24.1)	5.0 (2.5–8.0)	16.0 (10.0–24.1)	7.0 (4.0–24.0)	24.0 (4.5–24.1)
AUC_0–t_ (ng × h/mL)	92.0 (32)	68.4 (37)	85.1 (28)	66.7 (38)	105 (33)	73.5 (36)
*t*_1/2_ (h)	16.2 (14) (*n* = 21)	24.5 (12) (*n* = 10)	15.9 (15) (*n* = 14)	23.8 (12) (*n* = 8)	16.8 (13) (*n* = 7)	27.4 (9) (*n* = 2)

AUC_0−*t*_ area under the plasma concentration–time curve from time 0 to the time of the last quantifiable concentration, C_max_ maximum plasma concentration, CV coefficient of variation, EM extensive metabolizer, HTBZ dihydrotetrabenazine, IM intermediate metabolizer, NC not calculable, *t*_1/2_ half-life, *t*_max_ time of maximum plasma concentration

^a^*n* = 23, except where indicated

^b^*n* = 15 except where indicated

^c^*n* = 8 except where indicated

^d^Median (min–max)

## Discussion

Deutetrabenazine was rationally designed using the process of deuteration with the intent of slowing the CYP2D6-dependent breakdown of the active metabolites of tetrabenazine to their less potent O-desmethyl metabolites. Earlier studies confirmed that deutetrabenazine tablets administered with food can provide the desired overall exposure and a lower C_max_ with half the dose of tetrabenazine [5, 6] and a linear PK of the active metabolites. The study described herein investigated the effect of the strong CYP2D6 inhibitor paroxetine on the pharmacokinetics and safety of a single dose of deutetrabenazine.

Paroxetine administered with tetrabenazine increased C_max_ by 1.3- and threefold and AUC by 2.4- and ninefold for α-HTBZ and β-HTBZ, respectively [7, 9]. Based on these findings, it is recommended that patients who take strong CYP2D6 inhibitors and or CYP2D6 poor metabolizers (PM) limit their daily tetrabenazine intake to 50 mg, 50% of the maximum allowed daily dose (100 mg) [7, 9].

Deuterated α-HTBZ and β-HTBZ, the metabolites of deutetrabenazine, are also metabolized by CYP2D6 but at a lower rate [5]. Therefore, a weaker effect of the strong CYP2D6 inhibitor paroxetine on the CYP2D6 catalyzed metabolism would be expected, and indeed was confirmed in the present study for both active deuterated metabolites with a resultant increase for α-HTBZ of 1.2-fold for C_max_ and 1.8-fold for AUC_0–∞,_ and for β-HTBZ of 2.1-fold for C_max_ and 5.6-fold for AUC_0–∞_. The higher effect of impaired CYP2D6 functions on β-HTBZ compared to α-HTBZ is consistent for deutetrabenazine and tetrabenazine and suggests that α-HTBZ is not exclusively metabolized by CYP2D6. The reduced exposure to O-desmethyl metabolites of deuterated α-HTBZ and β-HTBZ confirmed the effect of the strong CYP2D6 inhibitor on the demethylation of α-HTBZ and β-HTBZ, which is catalyzed by CYP2D6.

Although there was considerable overlap in individual subjects, the effect of paroxetine was observed to be greater in subjects with extensive CYP2D6 metabolizer (EM) phenotype than those with and intermediate (IM) phenotype for both C_max_ and AUC. However, the minor differences between EM and IM do not demand a different dosing scheme for these populations. Maximum exposure to α-HTBZ and β-HTBZ is slightly higher in IM compared to EM in the absence of paroxetine but comparable between IM and EM in the presence of the inhibitor.

One of the limitations of the present study was that CYP2D6 poor metabolizer (PM) phenotype was not investigated. However, the effect of paroxetine has been shown to shift the CYP2D6 phenotype from EM to PM [12, 13], although not necessarily in 100% of those treated subjects [14]. Thus, the dose recommendations for those taking strong CYP2D6 inhibitors apply to PM as well. Conversely, the ultrametabolizer (UM) phenotype was also not assessed; however, the results by Laine et al. 2001 suggest that there are no safety concerns and that use of paroxetine would normalize the UM phenotype [15].

Safety data including ECG assessments did not indicate increased risk for side effects in the presence of a strong inhibitor at the tested dose. In previous clinical safety and efficacy studies, deutetrabenazine showed a favorable side effect profile that is overall similar to placebo [16]. These data are consistent with the moderate increase in maximum exposure and the generally lower C_max_ of the deuterated drug compared to the non-deuterated from previous reports [5, 6]. Although paroxetine substantially increased α-HTBZ and β-HTBZ exposure after administration of deutetrabenazine, the magnitude of the effect was less than that observed for tetrabenazine in a study with a similar design [7, 9]. Thus, these results indicate that deuteration reduces the impact of concomitant use of a strong CYP2D6 inhibitor on α-HTBZ and β-HTBZ exposure.

The information on the effects of impaired CYP2D6 metabolism of deutetrabenazine from this study together with the data from PK comparisons to tetrabenazine [5, 6] and population PK analyses (data on file, Teva Pharmaceutical Industries Ltd) were used to establish the doses in the phase 3 efficacy and safety studies which supported registration of deutetrabenazine (Austedo) as a treatment for chorea for Huntington’s disease and tardive dyskinesia [1, 3, 4]. Accordingly, the maximum recommended deutetrabenazine dose for patients with Huntington’s disease or tardive dyskinesia who have impaired CYP2D6 function (PM or taking strong CYP2D6 inhibitors) is 36 mg/day (18 mg BID), a 25% reduction in the maximum recommended dose for patients with normal CYP2D6 function, 48 mg/day (24 mg BID).

## Supplementary information

Below is the link to the electronic supplementary material.

Supplementary file1 (DOCX 323 KB)

## Data Availability

Qualified researchers may request access to patient-level data and related study documents including the study protocol and the statistical analysis plan. Requests will be reviewed for scientific merit, product approval status, and conflicts of interest. Patient-level data will be de-identified, and study documents will be redacted to protect the privacy of trial participants and to protect commercially confidential information. Please email USMedInfo@tevapharm.com to make your request.

## Code availability

(software application or custom code) n/a.
