# Supplementary material for: The effect of potent CYP2D6 inhibition on the pharmacokinetics and safety of deutetrabenazine in healthy volunteers
Source: Eur J Clin Pharmacol. 2021 Sep 1;78(1):11–8. doi: 10.1007/s00228-021-03202-0 (PMC8724172; doi:10.1007/s00228-021-03202-0)
Supplement: Supplementary file 1 — Supplementary file1 (DOCX 323 KB) [file 228_2021_3202_MOESM1_ESM.docx]

Supplemental files

**Supplemental Figure S1.** Metabolic pathways of tetrabenazine and deutetrabenazine. RR enantiomer depicted

Figure 4 from Schneider et al. Deutetrabenazine Pharmacokinetics and Metabolism. Clin Transl Sci (2020) 13, 707–717. Used with permission.

**Supplemental Table S1**. CYP2D6 Genotype and Phenotype

| Subject Number | Allele 1* | Allele 2* | Major duplication of either allele | Phenotype |
| --- | --- | --- | --- | --- |
| 1 | 1 | 2 | No | Extensive metabolizer |
| 2 | 4 | 10 | No | Intermediate metabolizer |
| 3 | 4 | 10 | No | Intermediate metabolizer |
| 4 | 1 | 2 | No | Extensive (or normal) metabolizer |
| 5 | 2 | 41 | No | Extensive (or normal) metabolizer |
| 6 | 1 | 2 | No | Extensive (or normal) metabolizer |
| 7 | 2 | 41 | No | Extensive (or normal) metabolizer |
| 8 | 4 | 10 | No | Intermediate metabolizer |
| 9 | 1 | 9 | No | Extensive (or normal) metabolizer |
| 10 | 1 | 5 | No | Intermediate metabolizer |
| 11 | 4 | 10 | Yes | Extensive (or normal) metabolizer |
| 12 | 1 | 1 | No | Extensive (or normal) metabolizer |
| 13 | 4 | 10 | No | Intermediate metabolizer |
| 14 | 1 | 2 | No | Extensive (or normal) metabolizer |
| 15 | 1 | 2 | No | Extensive (or normal) metabolizer |
| 16 | 1 | 1 | No | Extensive (or normal) metabolizer |
| 17 | 1 | 2 | No | Extensive (or normal) metabolizer |
| 18 | 4 | 10 | No | Intermediate metabolizer |
| 19 | 4 | 10 | No | Intermediate metabolizer |
| 20 | 1 | 1 | No | Extensive (or normal) metabolizer |
| 21 | 1 | 1 | No | Extensive (or normal) metabolizer |
| 22 | 1 | 1 | No | Extensive (or normal) metabolizer |
| 23 | 4 | 10 | No | Intermediate metabolizer |
| 24 | 1 | 1 | No | Extensive (or normal) metabolizer |

Subject 011 was excluded from the primary pharmacokinetic analysis population on the basis that he was classified as having an indeterminate CYP2D6 phenotype that potentially could be outside the phenotypes specified in the inclusion criteria.

**Table S2.** Adverse Events by Treatment Period

| Adverse Events | Treatment Period | | | | |
| --- | --- | --- | --- | --- | --- |
|  | Deutetrabenazine Alone  Day 1 - 3 | Paroxetine Alone  Day 4 - 10 | Deutetrabenazine + Paroxetine  Day 11 - 14 | Follow-up  Day 15 - 41 | Overall |
| Subjects dosed, n (%) | 24 (100%) | 24 (100%) | 24 (100%) | 24 (100%) | 24 (100%) |
| Subjects with AEs, n (%) | 3 (13%) | 14 (56%) | 3 (13%) | 1 (4%) | 16 (67%) |
| Gastrointestinal Disorders | 0 (0%) | 5 (21%) | 0 (0%) | 0 (0%) | 5 (21%) |
| Gingival pain | 0 (0%) | 1 (4%) | 0 (0%) | 0 (0%) | 1 (4%) |
| Hypoaethesia oral | 0 (%) | 1 (4%) | 0 (%) | 0 (%) | 1 (4%) |
| Nausea | 0 (0%) | 2 (6%) | 0 (0%) | 0 (0%) | 2 (8%) |
| Paraesthesia oral | 0 (0%) | 1 (4%) | 0 (0%) | 0 (0%) | 1 (4%) |
| Swollen tongue | 0 (0%) | 1 (1%) | 0 (0%) | 0 (0%) | 1 (4%) |
| Vomiting | 0 (0%) | 1 (1%) | 0 (0%) | 0 (0%) | 1 (4%) |
| General Disorders and Administration Site Conditions | 0 (0%) | 5 (21%) | 1 (4%) | 0 (0%) | 5 (21%) |
| Chest discomfort | 0 (0%) | 1 (4%) | 0 (%) | 0 (0%) | 1 (4%) |
| Feeling abnormal | 0 (0%) | 1 (4%) | 0 (0%) | 0 (%) | 1 (4%) |
| Feeling hot | 0 (0%) | 1 (4%) | 1 (4%) | 0 (0%) | 2 (8%) |
| Local swelling | 0 (0%) | 1 (4%) | 0 (0%) | 0 (0%) | 1 (4%) |
| Sensation of foreign body | 0 (0%) | 1 (4%) | 0 (0%) | 0 (0%) | 1 (4%) |
| Infections and Infestations | 0 (0%) | 1 (4%) | 0 (0%) | 0 (0%) | 1 (4%) |
| Viral Infections | 0 (0%) | 1 (4%) | 0 (0%) | 0 (%) | 1 (4%) |
| Metabolism and Nutritional disorders | 0 (0%) | 3 (13%) | 0 (0%) | 0 (0%) | 3 (13%) |
| Decreased appetite | 0 (0%) | 3 (13%) | 0 (0%) | 0 (0%) | 3 (13%) |
| Musculoskeletal and Connective Tissue Disorders | 0 (0%) | 2 (9%) | 0 (0%) | 1 (4%) | 3 (13%) |
| Back pain | 0 (0%) | 1 (4%) | 0 (0%) | 0 (0%) | 1 (4%) |
| Muscle tightness | 0 (0%) | 1 (4%) | 0 (0%) | 0 (%) | 1 (4%) |
| Sensation of heaviness | 0 (0%) | 0 (0%) | 0 (0%) | 1 (4%) | 1 (4%) |
| Nervous System Disorders | 1 (4%) | 7 (29%) | 2 (8%) | 1 (4%) | 10 (42%) |
| Dizziness | 1 (4%) | 1 (4%) | 0 (0%) | 1 (4%) | 3 (13%) |
| Headache | 0 (0%) | 5 (21%) | 2 (8%) | 1 (4%) | 7 (29%) |

Adverse events are classified according to MedDRA Version 15.0. Deutetrabenazine 22.5 mg was administered on days 1 and 11; Paroxetine 20 mg was administered once-daily on days 4 -12. Followup after discharge occurred on days 14 – 30.
